# 

**Published:** 1906-09

**Authors:** 


SUBSCRIPTION $1.00

PUBLISHED MONTHLY.

### ATLANTA JOURNAL-RECORD OF MEDICINE.

SEP. 21. [illegible]

**Successor to Atlanta Medical and Surgical Journal, Established 1855, and Southern Medical Record, Established 1870.**

VOL. VIII. ATLANTA, GA., SEPTEMBER, 1906. No. 6

